# Comparative Analysis of Local Control Prediction Using Different Biophysical Models for Non-Small Cell Lung Cancer Patients Undergoing Stereotactic Body Radiotherapy

**DOI:** 10.1155/2017/1436573

**Published:** 2017-06-14

**Authors:** Bao-Tian Huang, Wu-Zhe Zhang, Li-Li Wu, Pei-Xian Lin, Jia-Yang Lu

**Affiliations:** ^1^Department of Radiation Oncology, Cancer Hospital of Shantou University Medical College, Shantou, Guangdong 515031, China; ^2^Department of Nosocomial Infection Management, The Second Affiliated Hospital of Shantou University Medical College, Shantou, Guangdong 515041, China

## Abstract

**Purpose:**

The consistency for predicting local control (LC) data using biophysical models for stereotactic body radiotherapy (SBRT) treatment of lung cancer is unclear. This study aims to compare the results calculated from different models using the treatment planning data.

**Materials and Methods:**

Treatment plans were designed for 17 patients diagnosed with primary non-small cell lung cancer (NSCLC) using 5 different fraction schemes. The Martel model, Ohri model, and the Tai model were used to predict the 2-year LC value. The Gucken model, Santiago model, and the Tai model were employed to estimate the 3-year LC data.

**Results:**

We found that the employed models resulted in completely different LC prediction except for the Gucken and the Santiago models which exhibited quite similar 3-year LC data. The predicted 2-year and 3-year LC values in different models were not only associated with the dose normalization but also associated with the employed fraction schemes. The greatest difference predicted by different models was up to 15.0%.

**Conclusions:**

Our results show that different biophysical models influence the LC prediction and the difference is not only correlated to the dose normalization but also correlated to the employed fraction schemes.

## 1. Introduction

Stereotactic body radiotherapy (SBRT) has emerged as a favorable treatment alternative for early stage non-small cell lung cancer (NSCLC) patients who are medically inoperable or unwilling to undergo surgery [1–4]. Recent reports have demonstrated that SBRT provides outcomes that are equivalent to surgery [5–7].

Although SBRT for NSCLC has achieved encouraging outcomes, the dose-response relationship for tumor control probability (TCP) has been an area of intense investigation in radiation oncology. Recently, several dose-response relationship models were developed and their high accordance with the clinical data was fully demonstrated [8–10]. However, the proposed models were generated from patients with different tumor stage or treated with inhomogeneous fraction schemes that were reported to be associated with local control (LC) [11–16]. Therefore, we speculate the LC prediction in these biophysical models is different for the patients treated with the same fraction scheme. To the best of our knowledge, little is known about the difference in LC predicting models and the problem should be further explored.

This study aims to find the difference of LC prediction among various biophysical models by comparing the 2-year and 3-year LC values calculated from the treatment planning data. Our result can provide essential guidance for clinical SBRT treatment of lung cancer.

## 2. Materials and Methods

### 2.1. Ethics Statement

The protocol was approved by the Ethics Committees of the Cancer Hospital of Shantou University Medical College. Because this is not a treatment-based study, our institutional review board waived the need for written informed consent from the participants. However, the patient information was kept anonymous to protect their confidentiality. The methods in the study were performed in accordance with the approved guidelines and regulations.

### 2.2. Patient Eligibility

Computed tomography (CT) simulation data for 17 patients previously diagnosed with primary stage I NSCLC were included in the study. The age of the patients ranged from 51 to 76 years old.

### 2.3. Immobilization and CT Scanning

The patients were immobilized in the supine position with a vacuum bag (Medtec Medical, Inc. Buffalo Grove, IL) or a thermoplastic mask (Guangzhou Klarity Medical & Equipment Co., Ltd, Guangzhou, China). All of the patients underwent respiratory-correlated four-dimensional computed tomography (4DCT) scans under the free breathing condition using a 16-slice CT (Philips Brilliance CT Big Bore Oncology Configuration, Cleveland, OH, USA). CT images were acquired at a 3 mm slice thickness during scanning. The CT images were then delivered to Eclipse treatment planning system (Version 10.0, Varian Medical System, Inc., Palo Alto, CA) for target delineating, organs at risk (OARs) contouring, and treatment planning and treatment plans evaluation.

### 2.4. Target Delineating and OARs Contouring

The internal target volume (ITV) was defined as the combination of the gross tumor volume (GTV) delineated on ten phases of the 4DCT scans under the pulmonary windows. To account for the set-up uncertainties and potential baseline tumor shift, a planning target volume (PTV) was created by adding a uniform 0.5 cm margin expansion to the ITV. For OARs contouring, the whole lung was limited to the air-inflated lung parenchyma, and the GTV and trachea/ipsilateral bronchus were excluded according to the Radiation Therapy Oncology Group (RTOG) 0915 report [17]. The chest wall (CW) was segmented from the corrected lung edges with a 2 cm expansion in the lateral, anterior, and posterior directions, excluding the lung volume and the mediastinal soft tissue [18–20]. If the 2 cm expansion extended outside the body, then the contour extended only to the external patient surface. To avoid cumbersome delineation of the entire CW, we defined it within a 3 cm limit in the head-to-feet direction from the PTV [19].

### 2.5. Treatment Planning

Five different fraction schemes of 1 × 30 Gy, 3 × 15 Gy, 4 × 12 Gy, 3 × 18 Gy, and 5 × 10 Gy were prescribed. 1 × 30 Gy represented 30 Gy in 1 fraction. Other fraction schemes could be defined in the same manner. The treatment was planned on the averaged 4DCT image using the Eclipse treatment planning system. All plans were designed on a TrueBeam LINAC with a 6 MV flattening filter free (FFF) photon beam and a maximum dose rate of 1400 MU/min. Treatment plans were created using dual partial arcs to prevent irradiation from injuring the contralateral lung. The collimator angles for all plans were set to 30° and 330° to minimize the contribution of the tongue-and-groove effect to the dose. Optimization was performed using the progressive resolution optimizer (PRO_10028) algorithm implemented in Eclipse 10.0. The optimizing objectives were adjusted to ensure the maximum dose was around 120% of the prescribed dose and centered in the GTV. Dose calculation was performed using the anisotropic analytical algorithm (AAA_10028) with a grid resolution of 1 mm while accounting for heterogeneity correction. All of the dose constraints and dose volume limits for critical organs should meet the criteria of the RTOG 0915 protocol and other publications [17, 21]. To investigate whether different dose normalization will influence the LC prediction in the models, two types of dose normalization were generated: (1) the maximum dose (*D*_max_) was about 120% of the prescribed dose and centered in the GTV (refer to *P*_120%_); (2) *D*_max_ was about 110% of the prescribed dose and centered in the GTV (refer to *P*_110%_).

### 2.6. LC Data Predicting

**Figure 1:**
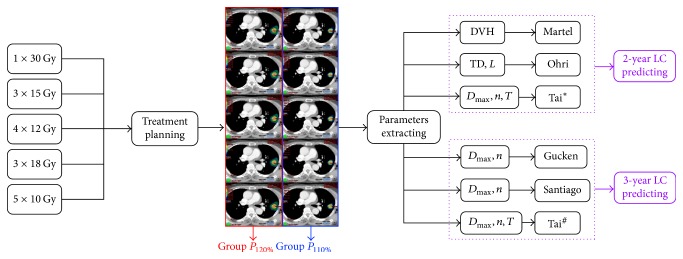
Flow chart of LC data prediction using different biophysical models. 1 × 30 Gy = 30 Gy in 1 fraction and other fraction schemes had similar definition. LC = local control. DVH = dose volume histogram. TD = treatment dose. *L* = diameter of the tumor. *D*_max_ = maximum dose in the target. *n* = fractions. *T* = follow-up time. *P*_120%_ = the maximum dose was 120% of the prescribed dose. *P*_110%_ = the maximum dose was 110% of the prescribed dose. ^**∗**^The Tai model for predicting the 2-year LC value. ^#^The Tai model for predicting the 3-year LC value.

The 2-year and 3-year LC data were predicted using 5 different biophysical models: the Martel model, the Ohri model, and the Tai model were used to calculate the 2-year LC data; the Gucken model, the Santiago model, and the Tai model were employed to calculate the 3-year LC value. All of the 5 models were generated from clinical data but each of them has their own characteristic. The Martel model, a parameterized dose-response characteristic using the logistic function, was conducted on 3-dimensional conformal radiation therapy (3DCRT) technique [22]. However, the model might potentially limit an appropriate valuation of LC prediction for patients undergoing intensity-modulated radiation therapy (IMRT) because of the superiority in delivering a more conformal dose and improving normal tissue sparing for IMRT compared with 3DCRT [23, 24]. The Ohri model which displayed a sigmoid-shaped dose-response curve between TCP and tumor size-adjusted biological effective dose (BED) was generated by retrospectively analyzing 504 NSCLC tumors treated with a variety of fraction schedules and was the unique model to take the effect of radiation dose and tumor size on LC into account to date [9]. Unfortunately, the model used the treatment dose for TCP prediction and thus the result was irrelevant to the dose irradiated on the tumor. Similar to the Ohri model, both of the Gucken and Santiago models also exhibited logistic relationship between TCP and the BED [8, 25]. However, they employed the isocenter dose, not the treatment dose as a predictor. The Tai model considered the tumor regrowth locally after radiation treatment and thus could be used to predict both the 2-year and 3-year LC data using isocenter dose [10]. The calculating process was performed using an in-house developed program on MATLAB 7.0 (MathWorks, USA). For 2-year LC estimation, *α*/*β*,* D*_50_, and*γ* were equal to 10 Gy, 72.0 Gy, and 2.0 for the Martel model [22]. *α*/*β*, *c*, TCD_50_, and *k* were taken as 10 Gy, 10 Gy/cm, 0 Gy, and 31 Gy, respectively, for the Ohri model [9]; for 3-year LC prediction, *α*/*β*, TCD_50_, and *k* were equal to 10 Gy, −1 Gy, and 80 Gy, respectively, for the Gucken model [8]. The same parameters were equal to 10 Gy, −60.2 Gy, and 113.3 Gy, respectively, for the Santiago model [25]. All of the modeling parameters in the Tai model for predicting 2-year and 3-year LC data were derived from multi-institution data fitting (Model Fit II) [10]. A flow chart of the radiobiological evaluation was presented in Figure 1.

### 2.7. Statistical Analysis

The differences of LC data between models were assessed by the Wilcoxon signed-rank test in two related samples using SPSS 19.0 (Chicago, IL). Difference was considered significant when *p* < 0.05.

## 3. Results

### 3.1. Patients' Characteristics

14 T1 (82.4%) and 3 T2 (17.6%) staging NSCLC patients were recruited in the study; 9 of them were male and the rest were female. Their median age was 65.3 ± 7.0 years old. The average tumor diameter and tumor volume were 2.5 ± 0.9 cm and 12.4 ± 17.4 cc, respectively. The patient characteristics were presented in Table 1.

### 3.2. Different Models Generate Completely Different LC Prediction

**Figure 2:**
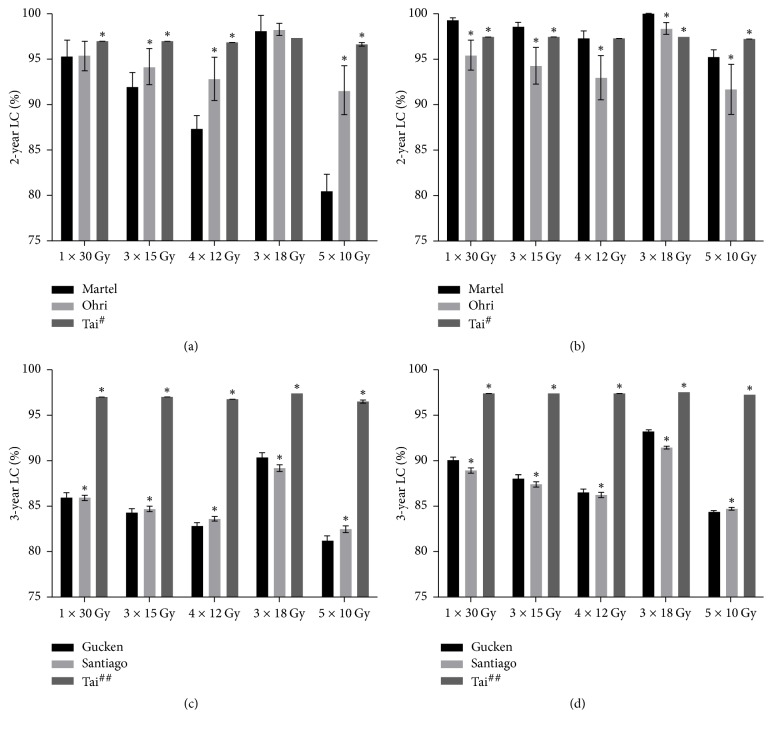
2-year and 3-year LC data in different predicting models. (a) 2-year LC data in the group *P*_110%_. (b) 2-year LC data in the group *P*_120%_. (c) 3-year LC data in the group *P*_110%_. (d) 3-year LC data in the group *P*_120%_. LC = local control. 1 × 30 Gy = 30 Gy in 1 fraction and so on. ^#^The Tai model for predicting the 2-year LC value. ^##^The Tai model for predicting the 3-year LC value. ^*∗*^Statistically significant with *p* value < 0.05 compared with the Martel and Gucken model for 2-year and 3-year LC prediction, respectively.

The 2-year and 3-year LC predictions in the five fraction schemes were displayed in Figure 2. Detailed difference among the models was shown in Table 2 (2-year LC data) and Table 3 (3-year LC data). It was found that different models resulted in completely different LC prediction. Exceptionally, the Gucken and the Santiago models exhibited quite similar 3-year LC value (Figure 2).

### 3.3. The Difference of 2-Year and 3-Year LC Prediction Is Correlated to Dose Normalization

As shown in Tables 2 and 3, the difference of 2-year and 3-year LC data was completely different in the group *P*_120%_ and group *P*_110%_ when the same fraction scheme was assigned. For 2-year LC predicting, the greatest difference was 15.6% versus 1.7% (Tai model, 5 × 10 Gy fraction scheme) in the groups *P*_110%_ and *P*_120%_, respectively, taking the Martel model as a reference. And it was 11.0% versus −2.6% for the Ohri model using the same fraction scheme. For 3-year LC predicting, the difference between the groups *P*_110%_ and *P*_120%_ was smaller than the 2-year data. The greatest difference was 10.7% versus 7.4% (Tai model, 1 × 30 Gy fraction scheme) in the groups *P*_110%_ and *P*_120%_, respectively, taking the Gucken model as a reference. The difference in any of the two models was statistically significant with* p* value < 0.05.

### 3.4. The Difference of 2-Year and 3-Year LC Prediction Is Associated with Fraction Schemes

The difference of 2-year and 3-year LC data was not only associated with the dose normalization but also associated with the employed fraction schemes. It was also found from Tables 2 and 3 that treatment plans prescribed with 5 fraction schemes exhibited totally different LC prediction. The 5 × 10 Gy fraction scheme displayed much greater differences compared with other fraction schemes. For 2-year LC predicting, the greatest difference was 15.6% versus 0.6% (Tai model, group *P*_110%_) in the 5 × 10 Gy and 1 × 30 Gy fraction schemes, respectively, using the Gucken model as a benchmark. For 3-year LC predicting, it was 12.9% versus 4.4% (Tai model, group *P*_120%_) in the 5 × 10 Gy and 3 × 18 Gy fraction schemes, respectively, using the Gucken model as a reference. The difference in any of the two models was also statistically significant with* p* value < 0.05.

## 4. Discussion

How great the difference is in various TCP-predicting models for NSCLC patients undergoing SBRT has not been well established. To address this issue, we employed 5 models generated from clinical data to predict the 2-year and the 3-year LC data for NSCLC patients. Our findings provide evidence that the predicted difference of 2-year and 3-year LC in biophysical models is not only associated with the dose normalization but also associated with the employed fraction schemes and different models influence the LC prediction by up to 15.0% (Tai model, group *P*_110%_). To the best of our knowledge, this is the first study investigating the influence of different biophysical models on LC prediction for NSCLC patients receiving SBRT.

We found that the difference of LC data using the 5 × 10 Gy fraction scheme was more remarkable than that from other fraction schemes. This finding might partly imply that the BED of the 5 × 10 Gy fraction scheme (BED_10_ = 100 Gy) probably lies in the steep region of the TCP-BED relationship curve, suggesting fraction schemes with higher BED_10_ are required to reach the asymptotic plateau to acquire stable TCP values. Although a calculated BED_10_ ≥ 100 Gy was generally reported to be associated with improved outcomes [12, 26], many clinical studies found the optimal dose for NSCLC patients undergoing SBRT exceeded 100 Gy, particularly when treating T2 stage patients [16, 27].

The difference of predicting data among the models may be caused by several reasons: (1) the models were derived from various tumor stage samples. Although all the 5 models were generated from stage I patients, different T1 and T2 portions might partly influence the modeling because higher dose is needed for T2 stage patients [16, 27], as mentioned before; (2) a wide variety of dose fractionation schemes were implemented for SBRT but the optimal one needed to be further determined. Fraction schemes with different BED are reported to influence the LC [13, 15]; (3) inhomogeneous dose prescription was used in these studies, such as 50% isodose covering 95% PTV, 100% isodose covering 95% PTV, and 60% isodose covering 100% PTV. These dose prescriptions make the isocenter dose vary from study to study and finally influence the model fitting.

In SBRT treatment for lung cancer, the dose is usually specified at the isocenter (denoted BED_ISO_) as well as at the PTV encompassing dose (denoted BED_PTV_). In the past few years, two independent studies tried to explore which dose normalization (BED_ISO_ and BED_PTV_) was more accurate to predict the LC for NSCLC patients undergoing SBRT [8, 25]. Consistently, both studies concluded that the BED_ISO_ was better to correlate with LC compared with the BED_PTV_. It is very reasonable to use the BED_ISO_ dose as the predictor because other more accurate algorithms, such as Acuros XB, mainly influence the dose in lung range but not the dose in the tumor [28–31]. To use the BED_ISO_ dose helps to eliminate the effect of dose difference to the GTV induced by different algorithms. However, how high the BED_ISO_ should be is not clearly defined in the dose specification of RTOG 0915 report. To distinguish how the BED_ISO_ dose influences the LC prediction, we employed two dose prescriptions, *P*_120%_ and *P*_110%_, in which the maximum dose (also denoted as isocenter dose) was about 120% and 110% of the prescribed dose. We find that group *P*_120%_ displays smaller difference among the predicting models than group *P*_110%_, indicating higher BED_ISO_ dose is recommended for SBRT treatment.

The employed Martel, Gucken, Santiago, and Tai model ignore a common intuition that the tumor size may influence the LC. However, it is noteworthy that whether the tumor size really matters is controversial nowadays. Some studies reported that tumor stage had statistically significant effect on LC for NSCLC patients undergoing SBRT [11, 14]; however, the point of view has been challenged by other investigations which showed that LC was not associated with tumor stage [32–34]. The contradiction is mainly induced by the employment of the risk-adapted prescribed dose fractionation based on tumor size in Baba and Allibhai's study [33, 34]. To date, none of the proposed models have taken the effect of the isocenter dose and tumor size on LC into account simultaneously, and a more comprehensive model is highly desired to fully interpret their impact.

Many retrospective studies have been conducted to explore the outcomes of SBRT treatment for NSCLC. Solda et al. analyzed 3771 patients with stage I NSCLC and found the average 2-year LC was 91% [5]. Zhang et al. enrolled 1102 stage I NSCLC patient in a meta-analysis and reported the 2-year LC is 92.3% [35]. The result predicted by the Ohri model was very close to that from two aforementioned studies, suggesting the model was more applicable for predicting the 2-year LC than the Martel and Tai models. As to the 3-year LC data, Guckenberger et al. found the 3-year LC was 83% in 159 pulmonary lesions [8]. Kestin et al. enrolled 505 T1 and T2 tumors treated at 5 different institutions and concluded that the 3-year LC was 91% [13]. Shibamoto et al. enrolled 180 patients with tumors that measured <1.5 cm, 1.5 to 3.0 cm, and >3.0 cm in greatest dimension and gave radiation doses of 44 Gy, 48 Gy, and 52 Gy, respectively. They found the 3-year LC rate was 86% for tumors ≤ 3 cm (44/48 Gy) and 73% for tumors > 3 cm [36]. The 5-year LC remained unchanged for the same patient cohort two years later [37]. The result that the 3-year LC data drops slightly from the 2-year LC predicted by the Tai model in our study (Figure 2) is highly in agreement with Shibamoto's result. Unfortunately, the estimated 3-year LC data from the Tai model was about 10% higher than the outcome obtained from the aforementioned references. On the contrary, both the Gucken and Santiago models predicted results similar to the clinical trials, indicating the Gucken and Santiago models were more applicable for predicting 3-year LC data than the Tai model.

The applicability of linear-quadratic (LQ) model during radiobiological evaluation is highly debated in recent years. Some investigators claim that the LQ model is applicable to SBRT. Guckenberger et al. suggested that the traditional LQ formalism could be accurately modeled compared to the LQ-L formalism for patients with stage I NSCLC undergoing SBRT based on 395 patients from 13 German and Austrian centers [8]. Shuryak et al. also found that LQ model provides significantly better fits to LC data for NSCLC than other models which required extra terms at high dose range did [38]. Santiago et al. analyzed 1975 patients to predict their 3-year LC and demonstrated that the LQ model could model local LC after hypofractionated irradiation and was a robust method for predicting clinical effects [25]. However, the aforementioned clinical evidence that supports the LQ model merely demonstrates that it does not significantly deviate from those expected from LQ model calculations and the data do not necessarily indicate that the LQ model fits best to the high dose data [39]. The inappropriateness of using the LQ model in SBRT is the overestimation of the effect at high fractional dose with no consideration of the phenomenon of reoxygenation during dose conversion. In using the LQ model, the correction of the errors was estimated at about 5–20% [39].

Although our study has demonstrated that different biophysical models do influence the LC prediction, there are some limitations. (1) It is important to note that the potential role of tumor hypoxia and reoxygenation is not explicitly considered in the 5 models used in the study. It is possible that the results may be different when incorporating the effect of them. Disappointedly, the effects of tumor hypoxia and reoxygenation during SBRT are not fully interpreted in modern radiation oncology. (2) The sample size of our study is a bit small to fully explain the differences in LC estimation among various biophysical models. Thus, a larger patient cohort is needed for further validation.

## 5. Conclusion

Our study demonstrates that different biophysical models influence the LC prediction for NSCLC patients undergoing SBRT. The differences should be carefully taken into account in clinical treatment and our results require further validation with larger sample size.

## Figures and Tables

**Table 1:** Basic information for 17 NSCLC patients undergoing SBRT.

Patient	Gender	Age	Stage^*∗*^
1	F	71	T1
2	M	71	T1
3	M	68	T1
4	F	72	T1
5	M	64	T1
6	M	68	T1
7	M	70	T1
8	M	62	T1
9	F	63	T1
10	F	70	T1
11	F	55	T1
12	M	62	T1
13	F	59	T1
14	F	76	T1
15	M	72	T2
16	F	56	T2
17	M	51	T2

M = male; F = female; ^*∗*^according to American Joint Committee on Cancer (AJCC), 7th edition.

**Table 2:** Difference of the 2-year LC data predicted using the Martel, Ohri, and Tai models.

FS	Group *P*_110%_			Group *P*_120%_		
	Median			Median		
	(Range)			(Range)		
	Martel	Ohri (%)	Tai (%)^#^	Martel	Ohri (%)	Tai (%)^#^
1 × 30 Gy	NA	−0.2 (−3~5.4)	0.6^†^ (0.6~6.4)	NA	−3.2^†^ (−8.6~−2.2)	−1.9^†^ (−2.0~−1.6)
3 × 15 Gy	NA	2.2^†^ (−2.1~7.2)	4.1^†^ (4.1~9.2)	NA	−3.5^†^ (−10.0~−1.4)	−1.4^†^ (−1.5~0.4)
4 × 12 Gy	NA	5.8^†^ (0~9.2)	8.6^†^ (7.7~12.2)	NA	−3.6^†^ (−11.3~−0.9)	−0.3 (−0.6~2.0)
3 × 18 Gy	NA	−0.5 (−1.5~4.7)	−1.6 (−2.1~3.9)	NA	−1.3^†^ (−3.4~−0.9)	−2.4^†^ (−2.5~−2.2)
5 × 10 Gy	NA	11.0^†^ (3.4~15.4)	15.6^†^ (14.6~19.6)	NA	−2.6^†^ (−11.5~0.2)	1.7^†^ (1.3~3.9)

The Martel model was taken as a benchmark in all data. FS = fraction scheme. *P*_120%_ = the maximum dose was 120% of the prescribed dose. *P*_110%_ = the maximum dose was 110% of the prescribed dose. 1 × 30 Gy = 30 Gy in 1 fraction and other fraction schemes had similar definition. NA = not available; ^#^the Tai model for predicting 2-year LC; ^†^statistically significant with *p* value < 0.05 compared with the Martel model.

**Table 3:** Difference of the 3-year LC data predicted using the Gucken, Santiago, and Tai models.

FS	Group *P*_110%_			Group *P*_120%_		
	Median			Median		
	(Range)			(Range)		
	Gucken	Santiago (%)	Tai (%)^#^	Gucken	Santiago (%)	Tai (%)^#^
1 × 30 Gy	NA	−0.2^†^ (−0.2~0.2)	10.7^†^ (10.7~11.9)	NA	−1.1^†^ (−1.3~−1.0)	7.4^†^ (6.8~8.1)
3 × 15 Gy	NA	0.3^†^ (0.3~0.7)	12.4^†^ (12.4~13.5)	NA	−0.7^†^ (−0.8~−0.5)	9.3^†^ (8.7~10.1)
4 × 12 Gy	NA	0.7^†^ (0.7~1.1)	13.7^†^ (13.6~14.5)	NA	−0.3^†^ (−0.5~0)	10.8^†^ (10.2~11.5)
3 × 18 Gy	NA	−1.3^†^ (−1.3~−1.0)	6.9^†^ (6.6~8.0)	NA	−1.7^†^ (−1.8~−1.7)	4.4^†^ (4.0~4.5)
5 × 10 Gy	NA	1.3^†^ (1.2~1.7)	15.1^†^ (14.9~15.9)	NA	0.4^†^ (0.3~0.4)	12.9^†^ (12.5~13.0)

The Gucken model was taken as a benchmark in all data. FS = fraction scheme. *P*_120%_ = the maximum dose was 120% of the prescribed dose. *P*_110%_ = the maximum dose was 110% of the prescribed dose. 1 × 30 Gy = 30 Gy in 1 fraction and other fraction schemes had similar definition. NA = not available; ^#^the Tai model for predicting 3-year LC; ^†^statistically significant with *p* value < 0.05 compared with the Gucken model.
